# Discoveries Interview: Prof. Garth L. Nicolson on cellular membrane structure

**DOI:** 10.15190/d.2013.2

**Published:** 2013-12-31

**Keywords:** Singer – Nicolson fluid-mosaic model

Over 40 years after the Singer – Nicolson fluid-mosaic model was launched, what have we learned?

**Figure 1:**
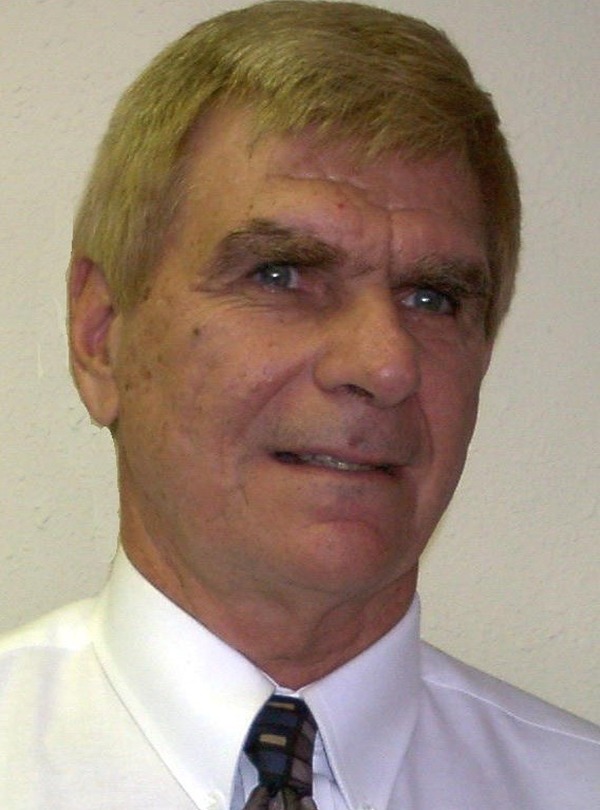
Professor Emeritus Garth L. Nicolson

**Professor Emeritus Garth L. Nicolson** is the *President,* *Chief Scientific Officer* and Research *Professor* at the Institute for Molecular Medicine in Huntington Beach, CA, USA. He is also a Conjoint Professor at the University of Newcastle (Australia).

He was formally the David Bruton Jr. Chair in Cancer Research and Professor and Chairman of the Department of Tumor Biology at the University of Texas M. D. Anderson Cancer Center in Houston, and he was Professor of Internal Medicine and Professor of Pathology and Laboratory Medicine at the University of Texas Medical School at Houston. He was also Professor of Comparative Pathology at Texas A & M University.

Professor Nicolson has published over 600 medical and scientific papers, including editing 19 books, and he has served on the Editorial Boards of 30 medical and scientific journals and was a Senior Editor of four of these. Professor Nicolson has won many awards, such as the Burroughs Wellcome Medal of the Royal Society of Medicine (United Kingdom), Stephen Paget Award of the Metastasis Research Society, the US National Cancer Institute Outstanding Investigator Award, and the Innovative Medicine Award of Canada. He is also a Colonel (Honorary) of the US Army Special Forces and a US Navy SEAL (Honorary) for his work on Armed Forces and veterans’ illnesses.

## **1. Can you describe in simple words the fluid-mosaic model of cellular membrane structure?**

The Fluid-Mosaic Membrane Model of cell membrane structure was the first model of the cell membrane to be based on thermodynamic principals and the available data on component lateral mobility within the membrane plane^1^. At the time, the existing membrane models were mostly a trilayer structure with extended proteins coating a phospholipid bilayer and a globular subunit structure without a phospholipid bilayer. However, neither of these latter models could reconcile the existing membrane lipid and protein data and the ability of many membrane components to rapidly move in the membrane plane. Thus, we proposed a model consistent with lipid bilayer features and inserted globular proteins into a liquid lipid bilayer so they could rapidly move in the membrane plane.

## **2. How was the model received by the scientific community? How has it evolved over time and what are the new advances?**

At the time it was published, the Fluid Mosaic Membrane Model was extremely well received by the science community, because it was consistent with existing data, especially new information on membrane protein and lipid mobility. In fact, the 1972 Science publication became one of the most cited publications in the biomedical sciences for many years. However, within a few years after its introduction, it was apparent to me that the original model was still valid but limited in terms of providing information about membrane linkages to the cytoplasm and information on domain structures in membranes^2^. These two areas brought new insights into controls over membrane dynamics and the mosaic properties of membranes. In recent years the documentation of specialized lipid domains that are important in cell signaling and the role of membrane barriers that reduce the freedom of mobility of membrane protein components have resulted in proposals on dynamic hierarchical membrane organization where membrane compartmentalization has made membranes more mosaic in structure compared to the original model^3^. Although the original model is still valid as a general scheme, it does not explain the many specialized structures (domains) in cellular membranes that actually limit the mobility and distribution of membrane components.

## **3. What will the field look like in 5-15 years?**

New data will continue to change the details on membrane dynamics and controls over membrane structures, but I doubt that the basic organization of cellular membranes at the nano-scale will change significantly. What will continue to change is that more detailed information on membrane signaling structures, membrane rearrangements and specialized membrane structures, their dynamics and their functional roles will be elaborated, and this will have to be incorporated into evolving models on membrane structure. In the last 40 years, we have seen tremendous advances in our knowledge of membranes, but there is still much to be learned, especially on cellular communication and how this fits into our evolving concepts of complex phenomenon, such as learning, memory and other aspects of animal and human interactions and how structural, dynamic and functional changes in membranes can be modified and even corrected in pathological situations.

## **4. What advices do you have for young researchers?**

I have always advised young scientists to follow their instincts into new exciting areas by incorporating and synthesizing information gathered from seemingly different disciplines. By working between different areas and incorporating information and techniques from different disciplines new progress and breakthroughs can be made on important problems. The one thing that I have always feared in the training of young researchers is that they are often educated mainly to continue in the same area of research using the same tools in which they have been trained. That is rarely how breakthroughs occur.

## **5. What are the most challenging, promising and/or the most rewarding areas of research in your opinion?**

There are too many to discuss here. But now is the time for basic researchers to answer questions that are not only important in, say, biology, chemistry or physics, but are also important in medicine, neurosciences and genetics, among others. The most challenging research areas at the moment incorporate elements of all of these.
